# Shifting mortality patterns of primary liver cancer in the United States: a 20-year analysis of disparities

**DOI:** 10.3389/fpubh.2025.1715729

**Published:** 2025-12-01

**Authors:** Haiyu Zhao, Lixing Ma

**Affiliations:** 1 Department of Gerontology, Changzhi People's Hospital, The Affiliated Hospital of Changzhi Medical College, Changzhi, Shanxi, China; 2 Department of Hepatobiliary Surgery, Changzhi People's Hospital, The Affiliated Hospital of Changzhi Medical College, Changzhi, Shanxi, China

**Keywords:** CDC WONDER, health disparities, primary liver cancer mortality, joinpoint regression, global disease burden

## Abstract

**Background:**

Primary liver cancer is a leading cause of cancer-related mortality worldwide. Despite its significant disease burden, long-term trends in primary liver cancer mortality across diverse demographic and geographic populations in the United States (U. S.) remain insufficiently characterized.

**Methods:**

We analyzed national mortality data from the Centers for Disease Control and Prevention Wide-ranging Online Data for Epidemiologic Research (CDC WONDER) database from 1999 to 2020. Deaths were identified using International Classification of Diseases, Tenth Revision (ICD-10) codes C22.0–C22.9, encompassing hepatocellular carcinoma (HCC), intrahepatic cholangiocarcinoma (ICC), and other specified or unspecified liver cancers. Age-adjusted mortality rates (AAMRs) were calculated and stratified by sex, ethnicity, census region, urbanization, and state. Temporal trends were assessed using joinpoint regression to estimate annual percent change (APC) and average annual percent change (AAPC).

**Results:**

Between 1999 and 2020, AAMRs increased steadily from 9.96 to 14.52 per 100,000 in males and from 4.56 to 6.35 per 100,000 in females, with consistently higher rates in males. Ethnic disparities were evident: non-Hispanic White (NH White) individuals had the lowest AAMRs but showed significant long-term increases (AAPC = 2.02%), whereas non-Hispanic Other (NH Other) populations experienced a decline (AAPC = −1.05%). Hispanic and non-Hispanic Black (NH Black) populations maintained persistently elevated mortality. Geographically, the South and West exhibited the highest AAMRs, although modest declines emerged in some regions after 2015. Both metropolitan and nonmetropolitan areas showed rising mortality, with steeper increases among nonmetropolitan populations. Substantial heterogeneity was observed across states, with most demonstrating positive AAPCs.

**Conclusion:**

Primary liver cancer mortality in the U. S. has risen markedly over the past two decades, with pronounced disparities by sex, ethnicity, region, urbanization, and state. These findings highlight the influence of sociodemographic and structural determinants on liver cancer outcomes. Efforts to reduce mortality should focus on equitable access to prevention, early detection, and treatment, particularly for high-burden populations.

## 1 Introduction

Primary liver cancer is the sixth most commonly diagnosed cancer and the third leading cause of cancer-related mortality globally (1). The burden of primary liver cancer has grown substantially over recent decades, driven by aging populations, persistent hepatitis B and C infections, increasing prevalence of alcohol-associated liver disease, and the rising epidemic of nonalcoholic fatty liver disease (2, 3). In the United States (U. S.), primary liver cancer incidence and mortality have escalated faster than many other malignancies, posing a growing public health challenge (4).

Despite this burden, long-term trends in primary liver cancer mortality across demographic and geographic subgroups remain incompletely characterized. Previous studies have largely focused on incidence or survival, with limited attention to mortality patterns that directly reflect the combined influence of prevention, detection, treatment, and comorbidities (5). Moreover, disparities in primary liver cancer outcomes by sex, ethnicity, socioeconomic status, and geography are increasingly recognized but not fully quantified at the national level. A comprehensive assessment of primary liver cancer-related mortality in the U. S. is therefore critical to identifying high-risk populations, informing public health strategies, and guiding healthcare resource allocation.

To address these gaps, we analyzed over two decades of national mortality data from the CDC WONDER. By examining temporal trends in AAMRs across demographic, regional, urbanization strata, and state, we aimed to provide an updated and detailed understanding of the evolving landscape of primary liver cancer mortality in the U. S.

## 2 Methods

### 2.1 Data sources

Mortality data for primary liver cancer (International Classification of Diseases, Tenth Revision [ICD-10] codes C22.0–C22.9) were obtained from the CDC WONDER database. These data provide nationally representative, population-based information on causes of death in the U. S., making them well suited for surveillance of long-term mortality trends. The study period spanned 1999–2020 and included underlying cause-of-death information from U. S. death certificates. Population denominators were derived from U. S. Census Bureau estimates to calculate mortality rates.

### 2.2 Study variables

We examined primary liver cancer–related deaths by sex, ethnicity (NH White, NH Black, Hispanic, and NH Other), census region (6), state, and urbanization status (metropolitan vs. nonmetropolitan, based on the 2013 NCHS Urban–Rural Classification Scheme) (7). These variables were selected to capture demographic and geographic heterogeneity that may contribute to disparities in liver cancer mortality.

### 2.3 Statistical analysis

AAMRs per 100,000 population were calculated using the 2,000 U. S. standard population to ensure comparability across demographic groups and over time. Given the descriptive and surveillance-oriented objectives of this study, trend analysis rather than causal modeling was prioritized. Temporal trends were assessed using the Joinpoint Regression Program (version 5.4.0; National Cancer Institute, Bethesda, MD, United States) (8, 9), which allows for the identification of statistically significant changes in trends over time. The maximum number of joinpoints was set to 4, and a log-linear model was used to estimate the APC and the AAPC, both with 95% confidence intervals (10). Statistical significance was defined as *p* < 0.05. Statistically significant values are marked with an asterisk (*) in the results section. Analyses were stratified by geographic variables to evaluate regional and urban–rural disparities in mortality patterns. This methodological framework was chosen to provide a robust, population-level description of mortality trends and disparities over time, consistent with the study’s surveillance purpose and the national public health relevance of primary liver cancer mortality.

## 3 Results

### 3.1 Primary liver cancer-related patterns in AAMR stratified by sex

**Figure 1:**
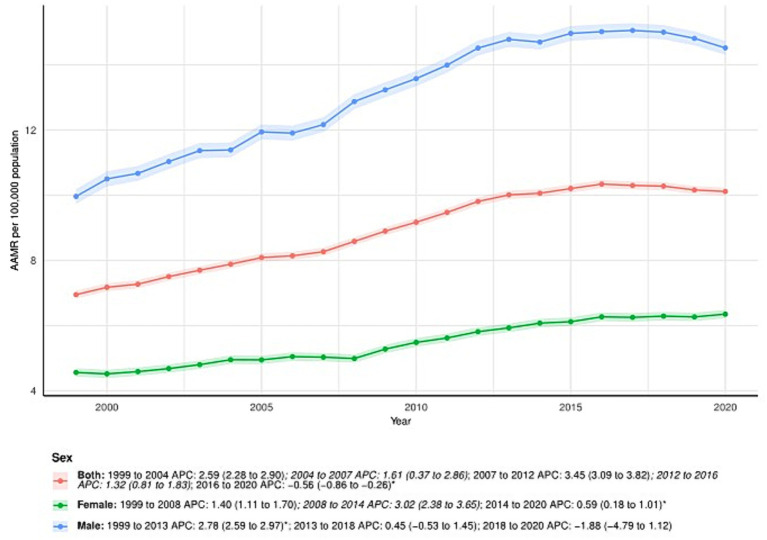
Sex-stratified deaths due to primary liver cancer: Age-adjusted mortality rates per 100,000 in the U. S., 1999–2020. APC: Annual percent change.

From 1999 to 2020, the AAMR in males increased from 9.96 (95% CI: 9.74–10.19) in 1999 to 14.52 (95% CI: 14.31–14.73) in 2020, with an AAPC of 1.77 (95% CI: 1.41–2.13). The APC for males varied across time periods: 1999–2013: APC = 2.78% (95% CI: 2.59–2.97); 2013–2018: APC = 0.45 (95% CI: − 0.53 to 1.45); 2018–2020: APC = −1.88 (95% CI: − 4.79 to 1.12). In female patients, the AAMR also increased over the study period, from 4.56 (95% CI: 4.43–4.70) in 1999 to 6.35 (95% CI: 6.22–6.48) in 2020. The AAPC was 1.63 (95% CI: 1.40–1.86). The APC by time period for females was: 1999–2008: APC = 1.40 (95% CI: 1.11–1.70); 2008–2014: APC = 3.02 (95% CI: 2.38–3.65); 2014–2020: APC = 0.59 (95% CI: 0.18–1.01). All the above changes are visualized in Figure 1 and further detailed in Supplementary Table S1.

### 3.2 Primary liver cancer-related patterns in AAMR stratified by ethnicity

Minor discrepancies were observed between the total number of subjects and the sum across ethnic groups in 1999 and 2020. Specifically, ethnicity information was missing for 31 subjects in 1999 and 70 subjects in 2020. The proportion of missing cases was minimal (<0.3%) and is unlikely to have influenced the observed temporal trends in age-adjusted mortality rates.

NH White consistently had the lowest AAMR among ethnic groups, increasing from 6.02 (95% CI: 5.89–6.15) in 1999 to 9.13 (95% CI: 9.00–9.27) in 2020, with an AAPC of 2.02 (95% CI: 1.82–2.22)*. APC by time period for NH White: 1999–2008: APC = 2.35 (95% CI: 2.22–2.49); 2008–2012: APC = 3.60 (95% CI: 2.95–4.26); 2012–2015: APC = 1.86 (95% CI: 0.69–3.06); 2015–2020: APC = 0.26 (95% CI: 0.02–0.51).

Among NH Other, AAMR decreased from 14.80 (95% CI: 13.76–15.83) in 1999 to 12.66 (95% CI: 12.10–13.22) in 2020. The AAPC was −1.05 (95% CI: −1.46 to −0.63). APC by time period: 1999–2013: APC = −0.33 (95% CI: −0.78 to 0.13); 2013–2020: APC = −2.47 (95% CI: −3.45 to −1.48).

The AAMR for NH Black increased from 9.27 (95% CI: 8.79–9.75) in 1999 to 12.01 (95% CI: 11.61–12.42) in 2020. The AAPC was 1.28 (95% CI: 0.87–1.69)*. APC by time period: 1999–2013: APC = 2.69 (95% CI: 2.48–2.91); 2013–2017: APC = 0.24 (95% CI: −1.52 to 2.03); 2017–2020: APC = −3.74 (95% CI: −5.41 to −2.05).

**Figure 2:**
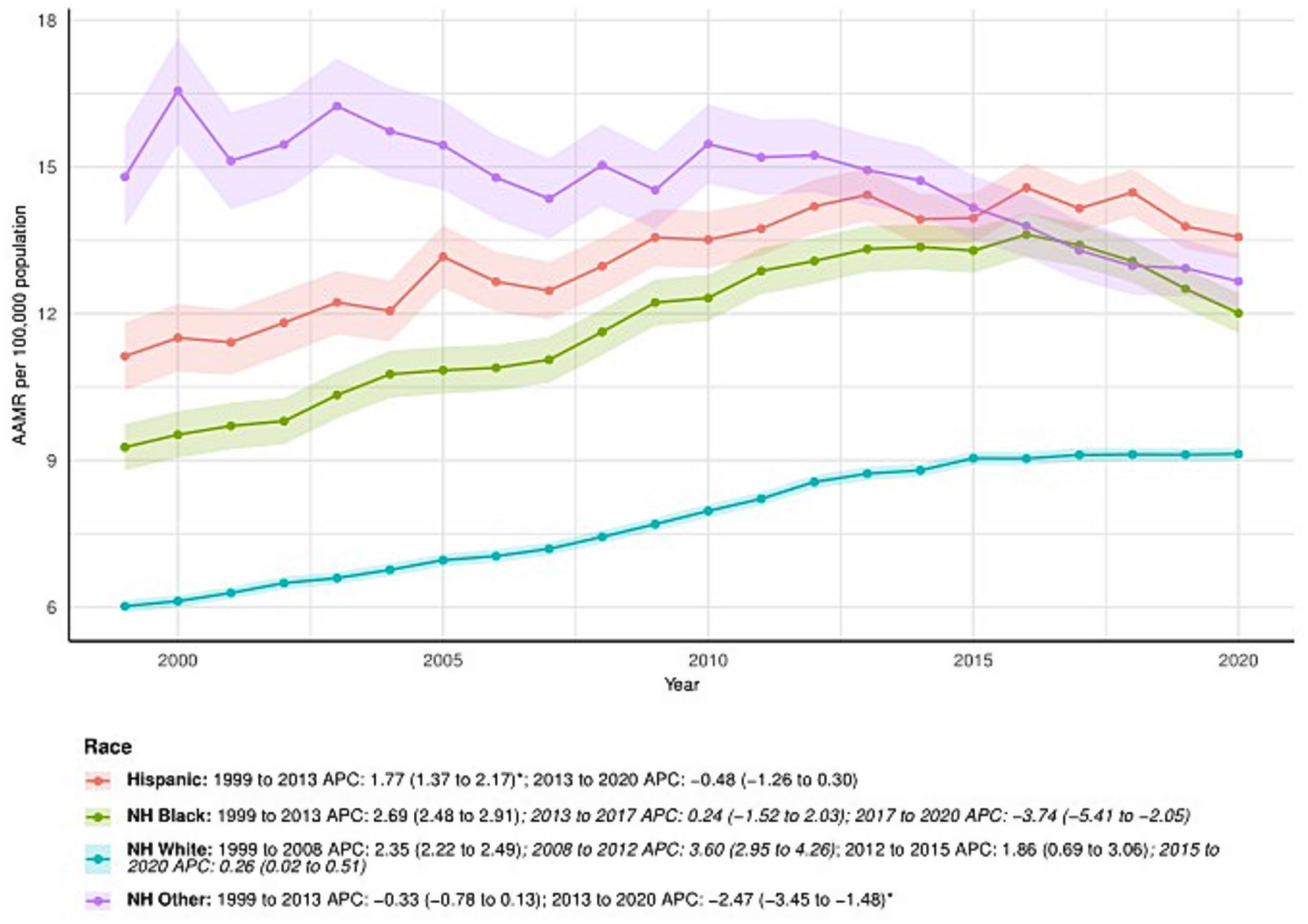
Ethnicity-stratified deaths due to primary liver cancer: Age-adjusted mortality rates per 100,000 in the U. S., 1999–2020. APC: Annual percent change.

Among Hispanic patients, the AAMR increased from 11.13 (95% CI: 10.43–11.83) in 1999 to 13.57 (95% CI: 13.12–14.01) in 2020. The AAPC was 1.01 (95% CI: 0.67–1.36). APC by time period: 1999–2013: APC = 1.77 (95% CI: 1.37–2.17); 2013–2020: APC = −0.48 (95% CI: −1.26 to 0.30). See Figure 2 and Supplementary Table S1 for detailed trends for all ethnic groups.

### 3.3 Primary liver cancer-related patterns in AAMR stratified by census regions

**Figure 3:**
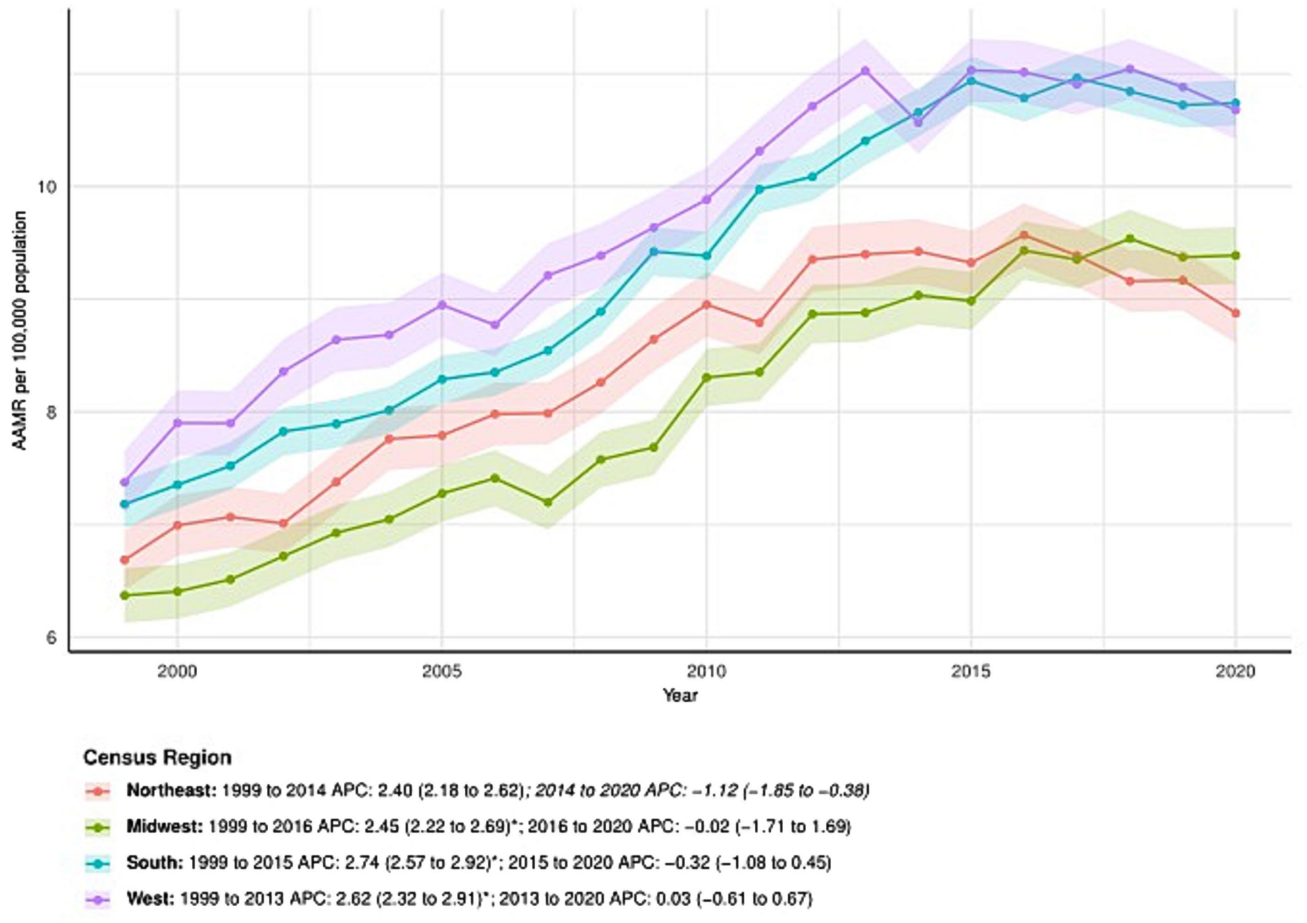
Census regions-stratified deaths due to primary liver cancer: Age-adjusted mortality rates per 100,000 in the U. S., 1999–2020. APC: Annual percent change.

All census regions showed an increase in AAMR over time, particularly in the South, which saw an increase from 7.18 (95% CI: 6.97–7.39) in 1999 to 10.74 (95% CI: 10.54–10.94) in 2020, with an AAPC of 2.01 (95% CI: 1.79–2.22). APC by time period for the South: 1999–2015: APC = 2.74 (95% CI: 2.57–2.92); 2015–2020: APC = −0.32 (95% CI: −1.08 to 0.45). The Northeast: 1999–2014: APC = 2.40 (95% CI: 2.18–2.62); 2014–2020: APC = −1.12 (95% CI: −1.85 to −0.38). The Midwest: 1999–2016: APC = 2.45 (95% CI: 2.22–2.69); 2016–2020: APC = −0.02 (95% CI: −1.71 to 1.69). The West: 1999–2013: APC = 2.62 (95% CI: 2.32–2.91); 2013–2020: APC = 0.03 (95% CI: −0.61 to 0.67). Regional differences are illustrated in Figure 3 and further detailed in Supplementary Table S1.

### 3.4 Primary liver cancer-related patterns in AAMR stratified by urbanization

**Figure 4:**
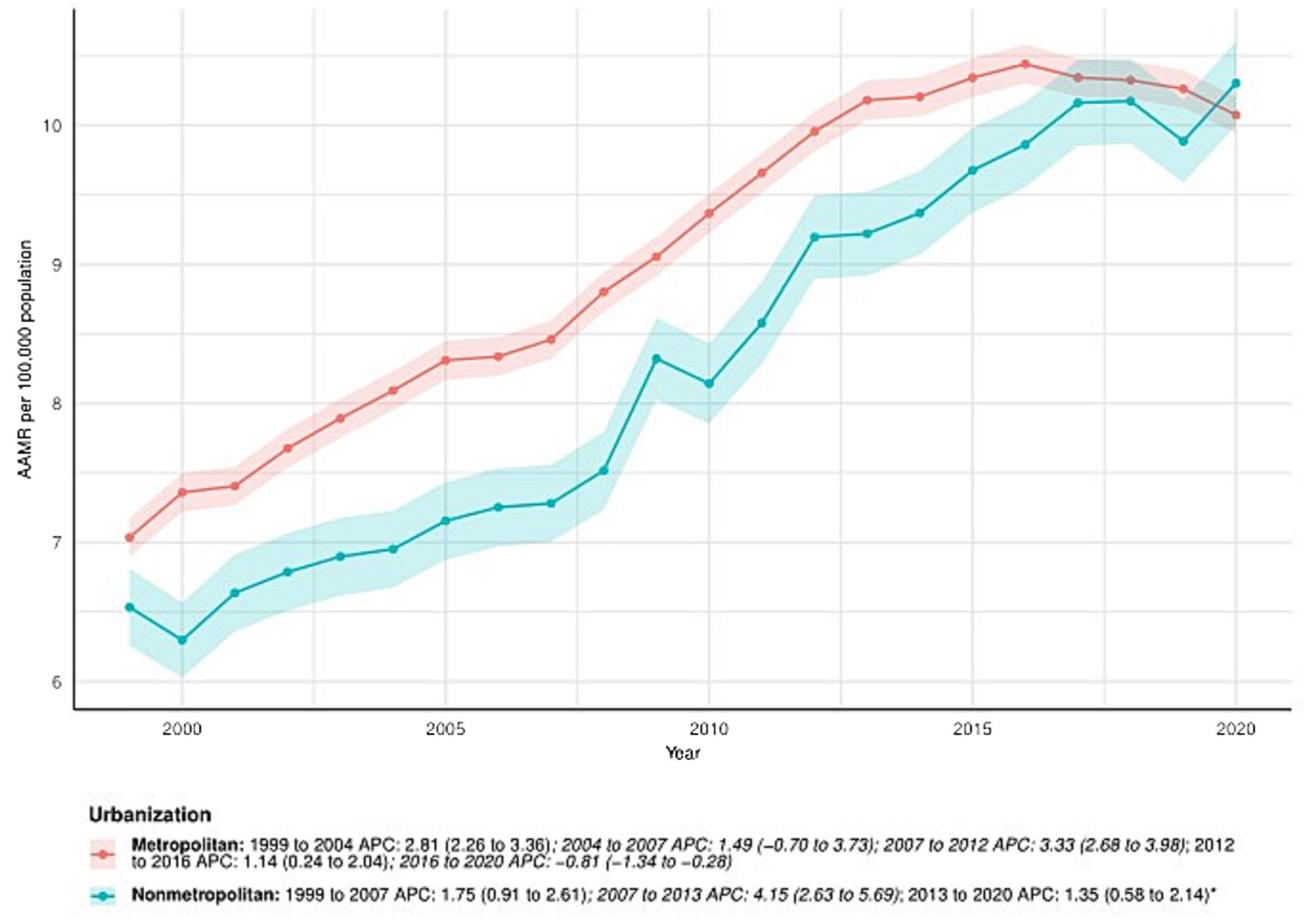
Urbanization-stratified deaths due to primary liver cancer: Age-adjusted mortality rates per 100,000 in the U. S., 1999–2020. APC: Annual percent change.

From 1999 to 2020, the AAMR in metropolitan areas increased from 7.04 (95% CI: 6.90–7.17) in 1999 to 10.08 (95% CI: 9.94–10.21) in 2020, with an AAPC of 1.73 (95% CI: 1.36–2.09)*. APC for metropolitan areas: 1999–2004: APC = 2.81% (95% CI: 2.26–3.36); 2004–2007: APC = 1.49 (95% CI: −0.70 to 3.73); 2007–2012: APC = 3.33 (95% CI: 2.68–3.98); 2012–2016: APC = 1.14 (95% CI: 0.24–2.04); 2016–2020: APC = −0.81 (95% CI: −1.34 to −0.28). In nonmetropolitan areas, the AAMR also increased, from 6.54 (95% CI: 6.26–6.81) in 1999 to 10.30 (95% CI: 10.00–10.61) in 2020. The AAPC was 2.30 (95% CI: 1.75–2.85). APC by time period: 1999–2007: APC = 1.75 (95% CI: 0.91–2.61); 2007–2013: APC = 4.15 (95% CI: 2.63–5.69); 2013–2020: APC = 1.35 (95% CI: 0.58–2.14). See Figure 4 and Supplementary Table S1 for details.

### 3.5 Distribution of state-level mortality and trends in the U. S.

**Figure 5:**
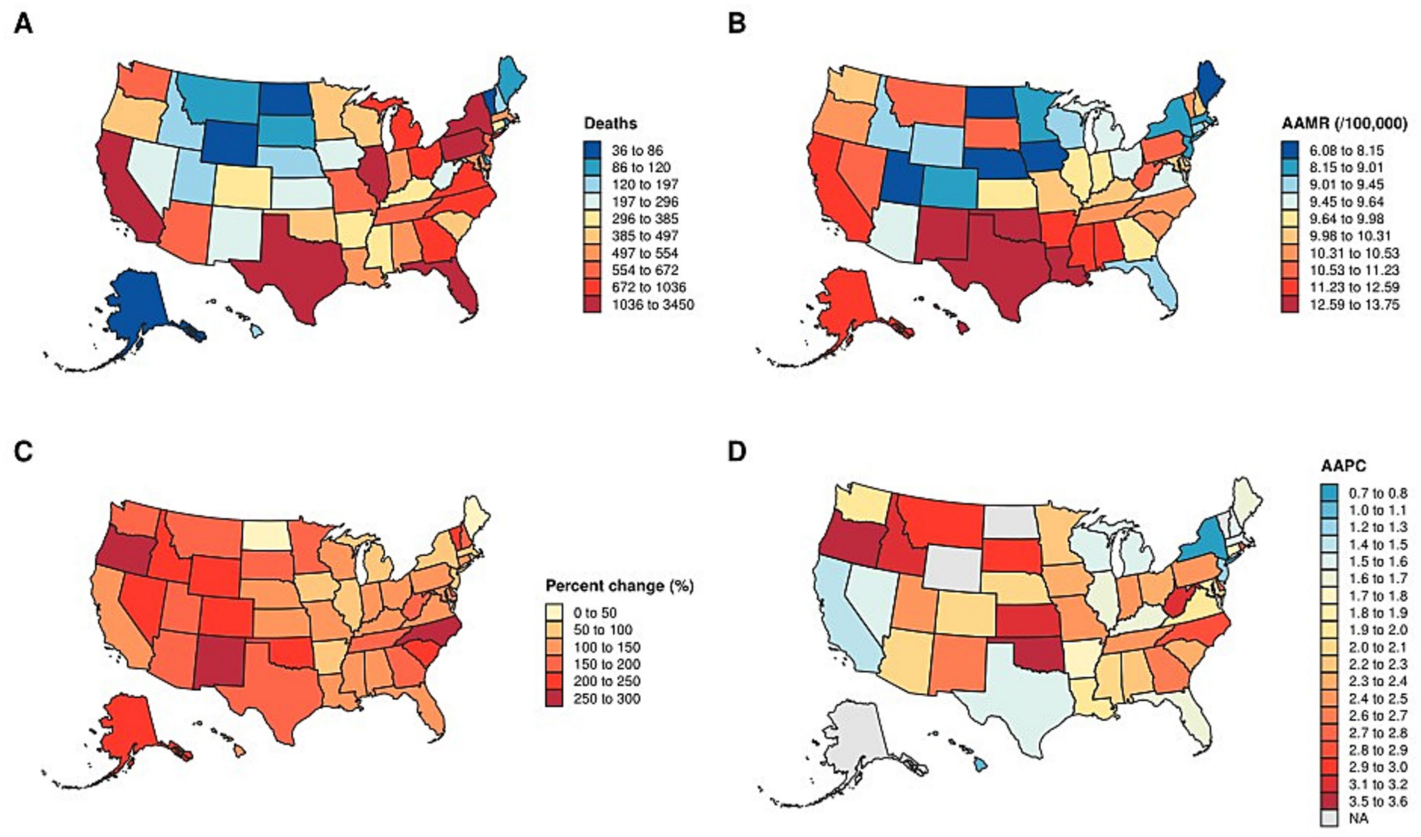
Distribution of state-Level mortality and trends in the U. S. **(A)** state-level total deaths in 2020. **(B)** AAMR in 2020. **(C)** percentage change in deaths from 1999 to 2020. **(D)** AAPC from 1999 to 2020. AAMR: Age-adjusted mortality rate. AAPC: average annual percent change.

In the spatial analysis, we mapped state-level total deaths (Figure 5A) and AAMR (Figure 5B) in 2020, as well as the percentage change in deaths (Figure 5C) and AAPC (Figure 5D) from 1999 to 2020. State-level distributions of deaths and AAMR were displayed using discrete classification with fixed legends, ensuring comparability across states. While some states reported the highest absolute number of deaths in 2020, their corresponding AAMR values were not always high, reflecting differences in population size and age structure (Supplementary Table S1). The percentage change in deaths over 1999–2020 was visualized with a warm color scale, highlighting substantial heterogeneity in growth magnitude across states. Most states experienced an increase, but the extent of change varied considerably. Finally, the distribution of AAPC was represented on a blue-to-red gradient, with negative values indicating a decline and positive values indicating an increase. Most states exhibited positive AAPC, consistent with a long-term upward trend (Supplementary Table S1).

## 4 Discussion

Primary liver cancer remains one of the most prevalent malignant tumors worldwide and represents a major public health challenge due to its high incidence and mortality (1). Despite its substantial global burden, important research gaps persist regarding primary liver cancer-related mortality trends, particularly across demographic and geographic subgroups (2, 11). To address this gap, we analyzed more than two decades of comprehensive CDC mortality data (1999–2020), providing an updated overview of long-term primary liver cancer mortality patterns in the U. S.

Unlike previous studies, our analysis is explicitly mortality-focused and covers the full ICD-10 C22.0–C22.9 spectrum. Adra et al. (12) analyzed long-term incidence and mortality trends using SEER-9, while Rehman et al. (13) examined only ICC with CDC WONDER data. Our study extends these efforts by focusing on mortality as the primary endpoint and integrating HCC, ICC, and other/unspecified C22 codes to capture the combined effects of prevention, detection, and treatment. We further stratify mortality by sex, ethnicity, census region, urbanization level (2013 NCHS classification), and state, creating a comprehensive disparity atlas that identifies where mortality gaps are greatest—an actionable, policy-relevant framework not previously available. By jointly presenting total deaths and AAMR (with standardized legends), mapping AAPC and percent change, and distinguishing burden from risk, we enhance interpretability and support evidence-based prioritization. More specifically, our findings reveal several noteworthy trends. First, primary liver cancer-related AAMRs steadily increased across census regions, particularly in the South (5). Consistent with prior literature, males exhibited higher mortality rates than females throughout the study period, suggesting sex-specific biological factors, behavioral and exposure patterns (e.g., alcohol consumption, smoking, viral hepatitis exposure), as well as disparities in disease management, may together contribute to outcome differences between males and females (14–18). Moreover, distinct ethnic, state and urban–rural disparities were identified, highlighting the complex interplay of socioeconomic determinants, healthcare access, and underlying risk exposures in shaping primary liver cancer outcomes. The observed deceleration or reversal in AAMR trends since approximately 2016 may reflect multiple converging factors. First, improvements in viral hepatitis prevention and management—including widespread adoption of direct-acting antivirals for HCV and expanded HBV vaccination coverage—have likely reduced the progression to cirrhosis and HCC in recent years (19, 20). Second, public health initiatives promoting reduced alcohol consumption and smoking cessation may have contributed to the slower increase or even decline in liver cancer mortality, particularly in urbanized and better-resourced regions (21). Third, the plateauing trends could reflect competing dynamics: decreasing viral hepatitis–related cases alongside a rising burden of metabolic liver disease, such as non-alcoholic fatty liver disease (NAFLD) and non-alcoholic steatohepatitis (NASH), which progress more slowly to advanced disease (3, 22). Lastly, the COVID-19 pandemic toward the end of the study period may have influenced mortality reporting and access to care, although its precise impact remains to be determined (23, 24).

The upward trend in primary liver cancer mortality may be explained by multiple converging factors (3). Improvements in diagnostic capacity, clinical awareness, and death certification practices likely contributed to more accurate recognition of primary liver cancer-related deaths over time (25). Additionally, population aging, coupled with the rising prevalence of chronic liver diseases—including viral hepatitis, alcoholic liver disease, and nonalcoholic fatty liver disease—likely contributed to the increased mortality burden among older adults (2, 3, 26). Structural inequities in healthcare access, particularly in underserved or rural communities, may further exacerbate mortality, as patients with primary liver cancer often face delays in diagnosis and barriers to effective treatment (27). Socioeconomic determinants such as poverty, low educational attainment, and unstable living conditions further compound these disparities (28). In addition, comorbidities—including metabolic disorders, cirrhosis-related complications, and cardiovascular disease—are frequently associated with primary liver cancer and may amplify the risk of adverse outcomes (29).

While this study provides valuable insights into long-term trends in primary liver cancer mortality across the U. S., several limitations merit consideration. Because the CDC mortality data are aggregated, potential misclassification and underreporting cannot be excluded (30), and the absence of individual-level information—such as antiviral treatment adherence, alcohol consumption, and lifestyle behaviors—precluded assessment of their specific contributions to mortality trends. Furthermore, the analysis did not evaluate the impact of therapeutic interventions on mortality nor distinguish among causes of death within the primary liver cancer population. Future research should extend this descriptive framework by linking population-based mortality data with detailed clinical, behavioral, and treatment information (e.g., SEER-Medicare or institutional cohorts) and incorporating spatial regression or multivariable modeling approaches to more precisely delineate the contextual determinants underlying the observed disparities (31–34).

From an epidemiological standpoint, males consistently show a higher baseline risk of primary liver cancer, with a reported male-to-female incidence ratio of approximately 2–3:1 (35). Consequently, increases in shared risk factors—such as alcohol consumption, obesity, and chronic viral hepatitis—tend to exert a stronger influence on men (36, 37). In contrast, females exhibit a lower overall risk, potentially due to the protective effects of estrogens and fewer harmful lifestyle exposures (16–18, 38). However, delayed diagnosis and sex-related differences in disease progression may also contribute to outcome disparities (39, 40). These patterns underscore the importance of integrating sex as a key determinant in liver cancer prevention and control strategies. Tailored interventions—such as reducing alcohol use, managing obesity, and improving screening for chronic hepatitis—are likely to produce greater benefits when adapted to the distinct risk profiles of men and women (41).

Building on these sex-specific insights, future research should aim to identify modifiable risk factors and assess how inequities in healthcare access affect management and survival, particularly among marginalized populations (3, 27). Evaluating the effectiveness of preventive and therapeutic measures—including hepatitis B vaccination, antiviral therapies for hepatitis B and C, lifestyle interventions, surveillance programs, and emerging systemic treatments—will be crucial for reducing mortality (42, 43). A refined understanding of evolving mortality trends will better equip policymakers, healthcare providers, and researchers to design targeted, evidence-based interventions that mitigate the growing burden of primary liver cancer and improve patient outcomes.

## 5 Conclusion

From 1999 to 2020, primary liver cancer-related mortality in the U. S. rose overall, with notable disparities by sex, ethnicity, region, urbanization, and state. Males, non-Hispanic Black, and Hispanic populations, as well as residents of the South and nonmetropolitan areas, bore disproportionately higher burdens. Although some subgroups have recently shown stabilization or modest declines, the persistent overall increase highlights the urgent need for targeted prevention, early detection, and equitable access to treatment to reduce disparities and curb the growing mortality burden.

## Data Availability

The original contributions presented in the study are included in the article/Supplementary material, further inquiries can be directed to the corresponding author.

## References

[1] Sung H Ferlay J Siegel RL Laversanne M Soerjomataram I Jemal A . Global cancer statistics 2020: GLOBOCAN estimates of incidence and mortality worldwide for 36 cancers in 185 countries. CA Cancer J Clin. (2021) 71:209–49. doi: 10.3322/caac.21660, PMID: 33538338

[2] Singal AG Lampertico P Nahon P. Epidemiology and surveillance for hepatocellular carcinoma: new trends. J Hepatol. (2020) 72:250–61. doi: 10.1016/j.jhep.2019.08.025, PMID: 31954490 PMC6986771

[3] Younossi ZM Koenig AB Abdelatif D Fazel Y Henry L Wymer M. Global epidemiology of nonalcoholic fatty liver disease—Meta-analytic assessment of prevalence, incidence, and outcomes. Hepatology. (2016) 64:73–84. doi: 10.1002/hep.28431, PMID: 26707365

[4] Ryerson AB Eheman CR Altekruse SF Ward JW Jemal A Sherman RL . Annual report to the nation on the status of cancer, 1975–2012, featuring the increasing incidence of liver cancer. Cancer. (2016) 122:1312–37. doi: 10.1002/cncr.29936, PMID: 26959385 PMC4840031

[5] White DL Thrift AP Kanwal F Davila J El-Serag HB. Incidence of hepatocellular carcinoma in all 50 United States, from 2000 through 2012. Gastroenterology. (2017) 152:812–820.e5. doi: 10.1053/j.gastro.2016.11.020, PMID: 27889576 PMC5346030

[6] United States Census Bureau Geographic levels. Available online at: https://www.census.gov/programs-surveys/economic-census/guidance-geographies/levels.html (2021)

[7] Ingram DD Franco SJ. 2013 NCHS urban-rural classification scheme for counties. Vital Health Stat 2. (2014):1–73. 24776070

[8] Surveillance Research Program, National Cancer Institute 2025 Joinpoint Regression Software (Version 5.4.0 – April 2025) [Computer software]. Available online at: https://surveillance.cancer.gov/joinpoint/

[9] Clegg LX Hankey BF Tiwari R Feuer EJ Edwards BK. Estimating average annual per cent change in trend analysis. Stat Med. (2009) 28:3670–82. doi: 10.1002/sim.3733, PMID: 19856324 PMC2843083

[10] Kim HJ Fay MP Feuer EJ Midthune DN. Permutation tests for joinpoint regression with applications to cancer rates. Stat Med. (2000) 19:335–51. doi: 10.1002/(SICI)1097-0258(20000215)19:3<>3.3.CO;2-Q 10649300

[11] Altekruse SF McGlynn KA Reichman ME. Hepatocellular carcinoma incidence, mortality, and survival trends in the United States from 1975 to 2005. J Clin Oncol. (2009) 27:1485–91. doi: 10.1200/JCO.2008.20.7753, PMID: 19224838 PMC2668555

[12] Adra S Alabrach Y Hashem A Mahmoud A Khalouf A El-Khapery A . Trends of primary liver cancer incidence and mortality in the United States: a population-based study over the last four decades. PLoS One. (2024) 19:e0309465. doi: 10.1371/journal.pone.0309465, PMID: 39236039 PMC11376511

[13] Rehman OU Hayat MS Shoaib MM Ahmad E Nadeem ZA Zain A. Trends and disparities in intrahepatic cholangiocarcinoma-related mortality in the United States from 1999 to 2020. J Gastrointest Cancer. (2025) 56:53. doi: 10.1007/s12029-024-01132-5, PMID: 39869219

[14] El-Serag HB. Epidemiology of viral hepatitis and hepatocellular carcinoma. Gastroenterology. (2012) 142:1264–73. doi: 10.1053/j.gastro.2011.12.061 22537432 PMC3338949

[15] Petrick JL Kelly SP Altekruse SF McGlynn KA Rosenberg PS. Future of hepatocellular carcinoma incidence in the United States forecast through 2030. J Clin Oncol. (2016) 34:1787–94. doi: 10.1200/JCO.2015.64.7412, PMID: 27044939 PMC4966339

[16] Nevola R Tortorella G Rosato V Rinaldi L Imbriani S Perillo P . Gender differences in the pathogenesis and risk factors of hepatocellular carcinoma. Biology (Basel). (2023) 12:984. doi: 10.3390/biology12070984, PMID: 37508414 PMC10376683

[17] Al Ta'ani O Al-Ajlouni Y Jagdish B Khataniar H Aleyadeh W Al-Bitar F . Examining the evolving landscape of liver cancer burden in the United States from 1990 to 2019. BMC Cancer. (2024) 24:1098. doi: 10.1186/s12885-024-12869-4, PMID: 39232707 PMC11373298

[18] Chen CL Kuo MJ Yen AM Yang WS Kao JH Chen PJ . Gender difference in the association between metabolic factors and hepatocellular carcinoma. JNCI Cancer Spectr. (2020) 4:pkaa036. doi: 10.1093/jncics/pkaa036 33134821 PMC7583157

[19] Polaris Observatory Collaborators. Global prevalence, treatment, and prevention of hepatitis B virus infection in 2016: a modelling study. Lancet Gastroenterol Hepatol. (2018) 3:383–403. doi: 10.1016/S2468-1253(18)30056-6 29599078

[20] Stanaway JD Flaxman AD Naghavi M Fitzmaurice C Vos T Abubakar I . The global burden of viral hepatitis from 1990 to 2013: findings from the global burden of disease study 2013. Lancet. (2016) 388:1081–8. doi: 10.1016/S0140-6736(16)30579-7, PMID: 27394647 PMC5100695

[21] Islami F Ward EM Sung H Cronin KA Tangka FKL Sherman RL . Annual report to the nation on the status of Cancer, part 1: National Cancer Statistics. J Natl Cancer Inst. (2021) 113:1648–69. doi: 10.1093/jnci/djab131, PMID: 34240195 PMC8634503

[22] Huang DQ El-Serag HB Loomba R. Global epidemiology of NAFLD-related HCC: trends, predictions, risk factors and prevention. Nat Rev Gastroenterol Hepatol. (2021) 18:223–38. doi: 10.1038/s41575-020-00381-6, PMID: 33349658 PMC8016738

[23] Cholankeril G Goli K Rana A Hernaez R Podboy A Jalal P . Impact of COVID-19 pandemic on liver transplantation and alcohol-associated liver disease in the USA. Hepatology. (2021) 74:3316–29. doi: 10.1002/hep.32067, PMID: 34310738 PMC8426752

[24] Gao X Lv F He X Zhao Y Liu Y Zu J . Impact of the COVID-19 pandemic on liver disease-related mortality rates in the United States. J Hepatol. (2023) 78:16–27. doi: 10.1016/j.jhep.2022.07.028, PMID: 35988691 PMC9611810

[25] Altekruse SF Henley SJ Cucinelli JE McGlynn KA. Changing hepatocellular carcinoma incidence and liver cancer mortality rates in the United States. Am J Gastroenterol. (2014) 109:542–53. doi: 10.1038/ajg.2014.11, PMID: 24513805 PMC4148914

[26] Rehm J Samokhvalov AV Shield KD. Global burden of alcoholic liver diseases. J Hepatol. (2013) 59:160–8. doi: 10.1016/j.jhep.2013.03.007, PMID: 23511777

[27] Nderitu P Bosco C Garmo H Holmberg L Malmström H Hammar N . The association between individual metabolic syndrome components, primary liver cancer and cirrhosis: A study in the Swedish AMORIS cohort. Int J Cancer. (2017) 141:1148–1160. doi: 10.1002/ijc.30818 28577304

[28] Jemal A Ward EM Johnson CJ Cronin KA Ma J Ryerson B . Annual report to the nation on the status of cancer, 1975–2014, featuring survival. J Natl Cancer Inst. (2017) 109:djx030. doi: 10.1093/jnci/djx030, PMID: 28376154 PMC5409140

[29] Dyson J Jaques B Chattopadyhay D Lochan R Graham J Das D . Hepatocellular cancer: the impact of obesity, type 2 diabetes and a multidisciplinary team. J Hepatol. (2014) 60:110–7. doi: 10.1016/j.jhep.2013.08.011, PMID: 23978719

[30] Murphy SL Xu J Kochanek KD Curtin SC Arias E. Deaths: final data for 2015. Natl Vital Stat Rep. (2017) 66:1–75. PMID: 29235985

[31] Henley SJ Ward EM Scott S Ma J Anderson RN Firth AU . Annual report to the nation on the status of cancer, part I: national cancer statistics. Cancer. (2020) 126:2225–49. doi: 10.1002/cncr.32802, PMID: 32162336 PMC7299151

[32] Elliott P Wartenberg D. Spatial epidemiology: current approaches and future challenges. Environ Health Perspect. (2004) 112:998–1006. doi: 10.1289/ehp.6735, PMID: 15198920 PMC1247193

[33] Kürüm E Nguyen DV Qian Q Banerjee S Rhee CM Şentürk D. Spatiotemporal multilevel joint modeling of longitudinal and survival outcomes in end-stage kidney disease. Lifetime Data Anal. (2024) 30:827–52. doi: 10.1007/s10985-024-09635-w, PMID: 39367291 PMC11502599

[34] Ali MS Alemu TG Techane MA Wubneh CA Assimamaw NT Belay GM . Spatial variation and determinants of underweight among children under 5 y of age in Ethiopia: a multilevel and spatial analysis based on data from the 2019 Ethiopian demographic and health survey. Nutrition. (2022) 102:111743. doi: 10.1016/j.nut.2022.111743, PMID: 35816812

[35] Yao Z Dai C Yang J Xu M Meng H Hu X . Time-trends in liver cancer incidence and mortality rates in the U.S. from 1975 to 2017: a study based on the surveillance, epidemiology, and end results database. J Gastrointest Oncol. (2023) 14:312–24. doi: 10.21037/jgo-23-25, PMID: 36915450 PMC10007921

[36] Devarbhavi H Asrani SK Arab JP Nartey YA Pose E Kamath PS. Global burden of liver disease: 2023 update. J Hepatol. (2023) 79:516–37. doi: 10.1016/j.jhep.2023.03.017, PMID: 36990226

[37] Calle EE Rodriguez C Walker-Thurmond K Thun MJ. Overweight, obesity, and mortality from cancer in a prospectively studied cohort of U.S. adults. N Engl J Med. (2003) 348:1625–38. doi: 10.1056/NEJMoa021423, PMID: 12711737

[38] Naugler WE Sakurai T Kim S Maeda S Kim K Elsharkawy AM . Gender disparity in liver cancer due to sex differences in MyD88-dependent IL-6 production. Science. (2007) 317:121–4. doi: 10.1126/science.1140485, PMID: 17615358

[39] Yang JD Hainaut P Gores GJ Amadou A Plymoth A Roberts LR. A global view of hepatocellular carcinoma: trends, risk, prevention and management. Nat Rev Gastroenterol Hepatol. (2019) 16:589–604. doi: 10.1038/s41575-019-0186-y, PMID: 31439937 PMC6813818

[40] Hassan MM Botrus G Abdel-Wahab R Wolff RA Li D Tweardy D . Estrogen replacement reduces risk and increases survival times of women with hepatocellular carcinoma. Clin Gastroenterol Hepatol. (2017) 15:1791–9. doi: 10.1016/j.cgh.2017.05.036, PMID: 28579181 PMC5901750

[41] Petrick JL McGlynn KA. The changing epidemiology of primary liver cancer. Curr Epidemiol Rep. (2019) 6:104–11. doi: 10.1007/s40471-019-00188-3, PMID: 31259140 PMC6599615

[42] Lok AS Zoulim F Dusheiko G Ghany MG. Hepatitis B cure: from discovery to regulatory approval. J Hepatol. (2017) 67:847–61. doi: 10.1016/j.jhep.2017.05.008, PMID: 28778687

[43] Llovet JM Kelley RK Villanueva A Singal AG Pikarsky E Roayaie S . Hepatocellular carcinoma. Nat Rev Dis Primers. (2021) 7:6. doi: 10.1038/s41572-020-00240-3, PMID: 33479224 PMC13537433

